# Suit for Mal-practice Involving the Surgery of the Hip Joint

**Published:** 1876-10

**Authors:** 


### MISCELLANEOUS.

### Suit for Malpractice involving the Surgery of the Hip-Joint. Condensed from the Notes of the Superior Court Stenographer.

This was a suit for $15,000 damages by Charles Bergman against William Volker, for alleged mal-practice in not detecting and properly treating a dislocation of the left hip-joint of the plaintiff. W. W. Lyon and Mr. Hubbell attorneys for plaintiff. Henry W. Box and L. L. Lewis for defendant.

The grounds of the complaint rested upon the theory that Bergman had a dislocation of the left hip upon the dorsum of the ilium, which dislocation resulted from being knocked down upon his left side by a wagon on Aug. 31, 1875; that Volker was called to attend him on the fourth day of Sept., 1875, and failed to discover at that time, or any subsequent time, a dislocation; that the dislocation remained undiscovered until May 18th, 1876, when it was diagnosed by Dr. Gay, and by him reduced on the twenty-sixth of May.

The defense claimed that there was no dislocation, but on the contrary, an intra-capsular fracture, which at an early date, before separation of the impacted fragments and shortening took place, might have been easily overlooked, and that the treatment adopted by the defendant, in a man of Bergman's age, viz.: rest in bed, was as good as any. They further claimed that the defendant should not be held liable for damages as he only made four or five visits, at the last of which he declined to assume further control of the patient, not understanding his case.

The first medical witness called by the plaintiff was Dr. C. C. F. Gay.

Dr. Gay. I have been a practicing physician and surgeon for a quarter of a century. I first became acquainted with Charles Bergman on the eighteenth of May, 1875. I was invited to see him with Dr. Loomis, of this city. I found Bergman in bed, lying upon his back. I examined him first for disease of the heart, liver or kidneys, to account for the dropsy in his lower extremities, but found none. His limbs were very much swollen and there was an eczematous affection of the skin from the ankles to the knees. I examined him in a sitting posture. He said he had been suffering with great pain. I examined him for a cause. Found the left leg entirely useless. He was unable to move it. On sight I pronounced it a dislocation of the hip. I observed that the left leg was much shorter than the right. It was inverted, the toe turning in upon the dorsum of the right foot. I ascertained that the foot could not be everted or turned out beyond a perpendicular. There was a general immobility of the limb. There was a prominence upon the back of the hip which could not be felt upon the other side. It was above and back of the natural position of the trochanter. I took a tape-line and made the ordinary measurements to detect shortening, viz.: from the anterior superior spinous process of the ilium to the malleolus on either side. These measurements showed a shortening of the limb. Then pointed out these signs to Dr. Loomis, who was my associate. These symptoms showed a dislocation of the hip. They are the usual symptoms to be found in a case of dislocation, "and a wayfaring man though a fool, would not err therein." With these signs present, the natural diagnosis would be dislocation of the hip. From the condition of the man could not tell how long he had had the dislocation; could tell whether it was a recent or ancient dislocation. The patient represented himself to me as a great sufferer at that time, and not able to sleep without an anodyne. His appearance indicated as much. This was the result of the dislocation. Judging from appearances don't think I am able to draw a reasonable conclusion as to the length of time the patient had been in this condition. In general terms, would say a long time. He told me he had suffered nine months. (Objected to and objection over-ruled.) If the indications I have named were apparent when a surgeon was first called in, he should have been able to discover the dislocation

(Objected to on the ground of incompetency. Objection overruled, and exception noted.) The indications referred to were quite as discoverable at the first. A dislocation upon the dorsum of the ilium is easily to be discovered by a competent surgeon. Upon being called to attend in such a case as this, the first duty of a physician is to administer chloroform and after examining the limb, proceed with the aid of assistants to reduce it, if it was found to be a dislocation. Taking a perfectly healthy man, an injury of this kind, with the suffering and confinement it entailed, causing a reduction of vitality, might account for the dropsical effusion in the limbs. The dislocation could not, I think, have produced symptoms of rheumatism. Discovered no rheumatic symptoms at my first visit.

*Cross-examined.* The dividing line between an ancient and recent dislocation is not easy to fix. The latter must have occurred within a day or two, the former at a more or less remote period, say several weeks. Physical suffering night and day with great mental anxiety, together with taking large doses of opium might possibly have such an effect on the secretions as to induce dropsy. More pain is consequent upon dislocation of the hip than upon fracture of the femur. I have known of cases of ancient dislocations of the femur, but have never had any under my personal supervision. Have treated recent cases. A dislocation of the hip may be produced by a heavy weight falling on a man's back, or by a direct blow from a falling body, or by the man himself falling over on his body. The same direct blow that in one case dislocates a hip, in another may fracture it. It would be more likely to produce a fracture. All fractures are not easily detected soon after injury. A fracture of the neck of the femur may not show shortening immediately. Have never seen partial fractures of the femur. Fractures of the neck of the femur in old people have been produced by muscular action alone. A case of a recent fracture within the capsular ligament without a separation of the bone, would be difficult to detect. There would be no shortening except through gradual absorption. Have known cases of fracture of the neck of the femur where, in several weeks, shortening began to take place. On Friday last (Oct. 13) saw the patient. There were present, upon my invitation, Drs. Winne and Barnes; Dr. Moore, of Rochester, the two Drs. Miner, Dr. White and Dr. Rochester, of this city, and Dr. Nichell, of California, were also present. Measurements of the patient's limb were taken by myself, Dr. Moore, Dr. White, Dr. Julius F. Miner, and Dr. Rochester. The latter did not declare the result of his measurement. I don't swear that I reduced the dislocation of the limb. I did manipulate the limb with a view to reducing it. I don't remember that I measured the limb previous to this. It was measured previously. Cannot swear positively, but think I am safe in saying it was by Drs. Burwell, Hauenstein, Diehl, Hopkins and Bartow. I took no part in their efforts, wishing each to decide for himself as to the diagnosis. Did not see their measurements. Have not reported the case. Have made a rough sketch of it. I claim there was a shortening of about two inches or two inches and a half when I went to reduce the dislocation. Asked the patient if he had ever noticed that one leg was shorter than the other. This was upon the occasion of my first visit to him. He said he had not. Asked him, I think, if he had ever suspected that his leg was dislocated. He said "No." I did not have a very fair opportunity of making any measurement on Friday, but will say that I found the shortening less than the average given by the others. I neglected to note down my measurement. You may call it an inch and a half, if you please. Dr. White's varied from $2\frac{3}{4}$ of an inch to 2 inches, Dr. Moore's was 2 inches, Dr. Miner's $2\frac{3}{8}$ inches, and $2\frac{5}{8}$. I will place mine at an inch and a half. The measurement of the injured limb from the major trochanter to the superior spinous process of the ilium by Dr. Moore, was $4\frac{3}{4}$ inches on one side, and $5\frac{1}{4}$ inches on the other. This would indicate dislocation of the hip. I suspect that the hip is now dislocated. Did report to the medical society in September last, orally, that I had reduced the dislocation. Presume that I have made the same statement to others. Have given no facts to any medical journal. Did claim before the American Medical Association in Philadelphia, that I thought I had reduced a dislocation of nine months' standing. My opinion as to the result of my manipulations has not been changed. Have suspected from time I was first called to see Bergman, that the head of the bone was out of the socket, and had taken plenty of time to determine this point. Have kept this to myself. The examination of council of physicians was after you (Mr. Box) had expressed a desire to have a council. Think I took Dr. Loomis, Dr. Winne, Dr. Wetmore and Dr. Bartow. Think that very likely I claimed I had reduced the dislocation. Did get them to subscribe to a paper relating to certain things, which was drawn up by Dr. Bartow, acting as my *amanuensis*. Did not hint at a suspicion that there was still a dislocation. Don't remember that I claimed a reduction in positive terms. I might have done so. Did claim that I had made the reduction before Dr. Miner, Dr. Rochester, and the other physicians. My language was, "that I had reduced the bone." Have been led to believe that there is still a dislocation by a more recent examination, made yesterday (Oct. 15). After the Bergman-McArthur trial, went to reduce the dislocation. Told the physicians Friday that I felt I had reduced the dislocation. After the attempted reduction I visited Bergman from 34 to 44 times. From the twenty-sixth of May last, up to the present time. Find the patient's leg still with a tendency to inversion. Did claim on Friday that the reduction had been accomplished by me, and accounted for the shortening by saying that I believed the head of the bone to have been absorbed. The difference caused by the tilting of the pelvis should be subtracted from the actual shortening of the limb. Found symptoms present yesterday (Oct. 15) to indicate a dislocation. My opinion now is that the man's hip is out of joint. Discovered the positive signs yesterday. The measurement yesterday from the major trochanter to the middle line of the body (the spine) showed a difference of ¾ of an inch; it was ¾ of an inch nearer the middle of the sacrum than on the right side. Another sign present was the arch formed by the lower portion of the spinal column. Do not think this would be found in a case of fracture of the neck of the femur. When the patient lies on his back with the limbs extended the arch is present. It disappears when the limbs are flexed. Think the head of the bone is now in the ischiatic notch. The limb with reference to motion is now nearly as perfect as it would be if the head were in its proper place in the acetabulum. If it were still on the dorsum of the ilium the limb would be shortened with a tendency to inversion more than at present. When the head is permanently removed from its natural position there is a tendency on the part of nature to fill up the acetabulum.

*Re-direct.* A physician of ordinary capacity ought to have discovered within a week or two that there was a fracture had one been present. When the neck of the femur is broken and retained in its normal position there would still be crepitus. I have never seen a partial fracture of the neck of the femur. In a case of impacted fracture the shortening might not be discovered for some weeks. It is generally harder to reduce an ancient dislocation than a recent one. It frequently happens in an attempt to reduce a dislocation from the dorsum of the ilium that the bone is put into the ischiatic notch, and it is always necessary to be very careful to avoid that. After my attempt at reduction the signs of dislocation were entirely removed. It looked as if the head of the head of the bone had slipped into the acetabulum. The legs were stretched down to an equal length. The left foot was naturally everted. In three days the pain and swelling began to abate.

*Cross-examined.* The signs of dislocation into the sciatic notch are not so prominent as upon the dorsum of the ilium. There would be shortening, but not so much. I did not discover any shortening after my manipulation. In stating my account of the operation last Friday I remarked that I made use of measures to prevent the bone from slipping into the ischiatic notch. Dislocations into this notch have been considered very difficult to reduce, and in the reduction of the dorsal dislocations every effort is made to prevent the accident. After the fourth or fifth attempt at reduction I had become very much exhausted, and in the last effort Dr. Bartow said we must not get this into the ischiatic notch. I then said: "Ischiatic notch or no, I shall rotate this limb inward and lodge it in the ischiatic notch rather than leave it where it is." I considered it better to do so or he would have been a cripple for life. No man can swear positively whether it is in the acetabulum or not. If it had been a fracture, instead of one and a half inches shortening he would have had a limb four inches short, because no pains had been taken to keep the limb from becoming short. The modern school of surgery approves of no extension in fracture of the neck in old persons. I am willing to swear that there was no fracture in this case.

*Re-direct.* When the bone has slipped into the ischiatic notch the surgeon is advised to let it remain there. No surgeon, in this case, would have been justified in making further manipulations. I do not wish to be misunderstood. I say I suspected from the time of the operation to this, that the head of the femur was in the ischiatic notch, but hoped it was in the acetabulum. No surgeon in the world would be able to detect a dislocation in this case without the closest scrutiny. All the symptoms of dislocation have disappeared except shortening. I do not wish the jury to think there are no signs that point to a possible reduction into the acetabulum, but I think there are unmistakable symptoms that it is in the ischiatic notch. I have looked at it from time to time and hoped it was in the acetabulum, and measured him to see if there was dislocation into the ischiatic notch, but when he was so comfortable I regarded it as good surgery, even in such a case, to let him entirely alone.

To Mr. Box. I have the authority of the first surgeon of the United States, Dr. Gross, of Philadelphia, to explain shortening after reduction. I have received a letter from him since going on the stand, and he tells me shortening can be accounted for in cases of ancient dislocation.

At this point the plaintiff rested.

The first witness for the defendant was Geo. Mesmer, who swore that he was present at the time of Volker's first visit to Bergman. That he assisted him out of bed, and that with his hand supporting him on the left side, Bergman was able to walk across the floor touching both feet to the floor; the toes, at least, of the left foot resting squarely on the floor. He complained of pain through the hips, and in the body in general.

Dr. Henry C. Frost. I am twenty-eight years of age. Graduated in Cleveland, afterward went to London, Eng. First saw Mr. Bergman in March last. Was called by Mr. Lyon. Made an examination for cause of dropsy. Found him in a sitting posture. He could not lie down on account of difficulty in breathing in that position. Complained of pain in his limbs. There was tenderness in region of right kidney, and over liver. Nothing attracted my attention to the hip. Saw nothing but the dropsy out of the way in the limbs. Again saw him in May last. My attention had been called to a claim of there being a dislocation of the hip. The patient was lying down in bed. Noticed an apparent shortening of the left limb. The foot was in its normal position.

The limb lay flat on its posterior surface. Could move it outward and inward. He complained of shooting pains in his limbs. On bending the thigh could not feel the head of the bone on the dorsum ilium, nor in any other position could I feel it. The limb was capable of making such movements as to exclude the hypothesis of dislocation.

*Cross-examined.* By Mr. Lyon. My diploma is dated in 1873. Told you (Mr. Lyon) that there was no dislocation of the hip, in a conversation we had together after my examination. There was some resistance when I flexed the limb. Can examine the lower part of the spinal curve by placing my hand upon it, without turning the patient over in bed. Was satisfied, after my last examination, that there was a fracture of the neck of the femur; attributed the dropsical affections to disease of the heart.

*Re-direct.* By Mr. Box. Fractures of the neck of the femur are more apt to occur in elderly people than dislocation. From the manner in which the accident occured to Mr. Bergman, and the violence of his fall, together with his age, I would say that it would be more more apt to produce a fracture than a dislocation.

Dr. John D. Hineman. Reside in Buffalo. Know Charles Bergman. Have treated him as a patient. Commenced to treat him first in February last. Had charge of him up to lately; two or three weeks ago. Remember the Bergman–McArthur trial. The last time I saw him, previous to the trial of that case, he was getting better. Treated him from February up to that time. He took to his bed about two months after I was first called. The pain in his limbs, especially the left, would not permit of his lying down at that time. Saw him the next day after he tried to kill himself. He was lying on the lounge at the time he attempted suicide. Told me he had cut a blood vessel and held a basin to catch the blood. When it was about full he fainted away, and the basin falling to the floor, alarmed the lady in the house and she coming in discovered his condition. I know nothing about his falling off of the lounge. After the operation by Dr. Gay, he was worse. I was sent for. Don't recollect who came for me. Think I was informed by a postal card. The pain was getting less and the dropsical affection becoming worse. Saw his limbs in bed frequently, before and after Dr. Gay was there. Never saw any inversion of the foot. If there had been any such thing, think I should have discovered it.

*Cross-examined.* By Mr. Hubbell. Mr. Bergman said he attempted suicide because he would rather die than live in such pain. I could not at first detect dislocation or fracture, in consequence of the dropsical swelling. He had also Eczema. Do not call dropsy a disease of itself, it is a symptom of a disease. In Bergman's case, after Dr. Gay performed the operation for reduction, it was some weeks before Bergman was as well a man as he is now. He was improving nicely previous to this, but had not gotten up out of his bed. Knew nothing about the operation by Dr. Gay. I was not consulted. Think I noticed slight crepitus. When I first saw Bergman, he was helpless to a considerable degree.

By Mr. Box. My patient was not benefited by Dr. Gay's operation. It was doubtful as to his recovery, for quite a while.

Dr. W. W. Miner. Am twenty-eight years of age. Have charge of the Siters of Charity Hospital, as surgeon. Dr. Julius F. Miner is my uncle. Was called to see Mr. Bergman Nov. 6th. Saw him at his home. He was sitting on a lounge. Made examinations necessary to answer my purpose, as a physician called to prescribe for a patient. Found dropsy of the limbs and and abdomen. He was suffering from the inconveniences of dropsy. Never saw a man whose tissues were so distended. He complained of pain and inability to lie in bed. Said he had met with an accident. He did not speak of pain in any particular part of his body other than the extremities. Endeavored to have him lie down on the lounge and straighten his limbs. It was impossible for him to do so. Looked for trouble with liver and secretions, to find cause of dropsical effusions. Both of his feet were on the floor. Noticed nothing abnormal in their position. Do not think it possible for a patient with a dislocated hip to sit in that position. From the history of the case I received and from my examination with the symptoms discovered, came to the conclusion that the man was suffering from extreme dropsical effusion. On another occasion went to see him with Dr. Julius F. Miner. Discovered no signs of dislocation or fracture. The first I heard of the trouble with hip was after the Bergman-McArthur trial. Visited him and found shortening of the left leg. Other signs of dislocation were absent. His position in bed was such that a man with dislocation could not assume. His knees were lying evenly together. There was no resistance to eversion. There was freedom from restratnt in this respect. Was satisfied that the condition was not that of dislocation.

Dr. Gay claimed, on Friday last, that there had been symptoms of dislocation, and that he had reduced it. Saw Mr. Bergman shortly after it was said Dr. Gay had claimed to have reduced the dislocation. Found he had an extension on his left leg. He at first refused to let me look at it. His daughter persuaded him to do so. The leg was shortened. Could see evidences of this as he lay in bed. Was present when measurements were made last Friday. After Dr. Gay's manipulations, as he lay in bed, his leg did not look as short, with the extension on. The extension is to preserve the natural length of the limb.

*Cross-examined.* By Mr. Hubbell. I have no permanent connection with the Medical College. Have no relation with my uncle in the way of business partnership. Do not office with him. Did not decide upon the cause of the dropsy at my first examination, but was satisfied of some organic disturbance. Neither myself nor Dr. Julius Miner knew of a fracture or a dislocation until Dr. Gay reported that he had reduced a dislocation. Last Friday I came to the conclusion that there was pretty strong evidences of a fracture of the neck of the femur.

Dr. Julius F. Miner. My attention was first called to the case of Bergman, Dec. 11th. Went to see him with my nephew. Found him sitting on a sofa, with his feet upon the floor, in an easy and natural manner, and both legs greatly distended. Asking for a history of the case no clew was given that afforded a satisfactory explanation of the cause of the dropsical effusion. Heard nothing about any injury to the man. Patient said very little and did not complain of one leg above the other. There was nothing seen in the appearance of the legs to indicate a fracture or dislocation. A dislocation upon the dorsum of the ilium throws one leg over so that one foot rests on the other. On Friday last laid patient on the table and measured from the anterior superior spinious process of the ilium down to the ankle joint. The most careful and critical examination to be made showed a shortening of two inches. The patient was somewhat more difficult to measure than a lean man. The shortening was located, by measurement from the major trochanter to the point of the ilium, in the neck of the femur, and not in the shaft. Found no evidence of dislocation upon the dorsum of the ilium or in the ischiatic notch. Other symptoms were mobility of the leg and smoothness of the joint surface, that there is no dislocation. Dr. Gay claimed in my presence on Friday last that he had reduced the dislocation. There is not more difficulty in detecting dislocation upon the dorsum of the ilium than in the ischiatic notch. It can be detected. I can swear that there was no evidence on Friday last of Bergman's having any dislocation in the ischiatic notch. The symptoms to indicate this are shortening and the presence of the head of the bone in the notch. The manner in which this patient was injured would not produce a dislocation. It would cause a fracture, an impacted fracture, or a fracture of the neck of the femur. Old men and women almost always fracture the neck of the thigh-bone when they fall down, instead of producing a dislocation. Dislocation is almost unheard of in such cases. There are cases of fracture of the neck of the femur where there is no laceration of the capsular ligament or injury to the periosteum. The natural tendency of the muscles in the cases of dislocation is to draw the bone up. There are cases of the fracture of the neck of the femur difficult to determine at first. A good surgeon is liable to fail to discover it. Shortening comes on slowly. The lounge Bergman sat upon when I saw him was low. Falls of a short distance sometimes fracture the bones of old people. On the night that he attempted to commit suicide a man being faint and coming down off the lounge with force, as it is said he did, and striking the trochanter without muscular intervention, a fracture of the femur might result.

*Cross-examined.* By Mr. Hubbell. Have known Dr. Volker fifteen or twenty years. He was one of my earliest acquaintances in Buffalo. He was then a laborer on Jefferson street. Left the case after examining for the cause of dropsical effusion, until last Friday, when, upon making an examination, I had no difficulty in determining that there was a fracture of the neck of the femur. Had no reason then, nor have I now, to suppose that the head of the bone had ever changed its position. Think it would be possible for a competent surgeon—an expert—to discover a fracture of the neck of the thigh-bone. He would, first, upon making the discovery, place the patient in bed, and in old people it would be safer and quite as well not to use splint, pull or dress them too much. and allow nature to take its own course. If the patient was suffering great pain, the best thing that could be done would be to give him relief with anodynes if he can bear them. Dropsy might follow from inactivity and feeble condition of circulation. If dislocation produced same condition of system, then dropsy would also ensue. If at my first examination I had known of an injury to the hip, and found a fracture or dislocation, then I might have accepted that as inducing the dropsical effusion. Drs. Moore, White, Rochester, Winnie, Gay, Barnes, Nichell, and W. W. Miner were present last Friday at the examination. If the patient did not tell the doctor about an injury when he called, the best surgeon might go away without discovering a fracture or dislocation, though it were there. If a man were suffering with great pain about the hip and limbs, a good surgeon should make a physical examination.

*Re-direct.* By Mr. Box. The weight of the body can be born upon the foot when the fracture is entirely within the capsule. The patient can also move about. Around the neck of the femur there is what is called a capsular ligament, which covers the head of the bone, and is so astonishingly strong, that use of the limb is permitted until inflammation sets in, when the soreness is such that immobility insues.

By Mr. Hubbell. When the head of the bone is thrown out of place, the socket might fill up, and the dislocation being reduced it is more apt to occur again, than at first.

Dr. Thomas F. Rochester. I assisted in examining Bergman on Friday last. In the first place we had a history of the case from Dr. Gay so far as he knew. He then described certain conditions he found and the inferences he drew therefrom. He said he had found dislocation upon the dorsum of the ilium, which he had reduced by manipulation. The patient was then placed upon the table and critically examined. The result was that shortening of the left leg from two to three and one-half inches, was found. I came to the conclusion that the man had no dislocation. The history of the case, the nature of the accident, the manner of the fall, the age of the person (this is very important), and the measurement, with the shortening, showed that there had been fracture of the neck of the thigh-bone.

The balance of Dr. Rochester's testimony was corroborative of that of Dr. Miner, and all the previous witnesses of the medical fraternity, called in the defense.

Dr. A. R. Wright testified that he had visited the patient February, 1876. That the position of the limbs was natural. That his attention was not especially called to any special troubles with the hip.

Testimony was also introduced at this point to show that the patient had not fallen from the the lounge, that accident having been spoken of as liable to cause the fracture.

Dr. Conrad Diehl. Am a physician and surgeon of ten years practice. Was present at the claimed or attempted reduction of the asserted dislocation. The patient was upon the lounge with both limbs extended and under chloroform. I had no history of the case, and as I came into the room, upon invitation, I examined the limb. The legs were both apart. There was no inversion of the injured limb, and but little of the foot. Dr. Gay, with assistance, proceeded to make his manipulations for reduction of dislocation, which he continued for about an hour.

The testimony of the defendant, William Volker, introduced at this time, showed that he was uneducated and wholly incompetent to practice medicine. He testified that he was called Sept. 4th, 1875, to see Bergman, to treat him for rheumatism. That he made an examination while he was standing up, and noticed nothing wrong in the position of the limbs or appearance of the hips. He was not asked at that time to take charge of the patient, and did not visit him until sent for at the expiration of about a week. Two or three more visits were paid to the patient, at the last of which he declined giving further attendance as he did not understand the case. As the patient was complaining still of soreness, he advised him to get on crutches, if possible. This was from two to three weeks after the injury.

Dr. James P. White gave a detailed account of the examination of the plaintiff by Drs. Miner, Moore, Rochester and himself, the commission appointed by the court. The plaintiff was first made to walk across the room, and when his back was turned he could recognize the deformity which would be produced by a fracture of neck of the femur. He made such measurements as to satisfy himself that the limb was over two inches short; measuring from the anterior superior spinous process of the ilium to the maleoli, on either side, these points being fixed by the thumbs of physicians present. He then measured from the trochanters on either side to the ankle joints, and the measurements agreed, showing that the shaft of the bones was not fractured. Measurements made by others present showed that the trochanter was one-half inch nearer the spine of the ilium than on the right side.

Manipulation of the limb, rotation, absence of inversion, etc., satisfied me that there was no dislocation, that the head of the femur was in the acetabulum, but that the neck had been fractured. In intra-capsular. and especially impacted fracture, the shortening is not at once made very manifest. I recently saw a case in Oneida county which illustrates this. Dr. Brush was with me at the time. A lady fell injuring her hip, but no displacement was detected. In the course of some weeks, three or four, shortening began to show itself, and the family were in inclined to blame their physician for not detecting it in the first place. He was a well informed practitioner, and had carefully watched the case.

Dr. White's testimony, which was given in careful detail, was coroborative of the other medical witnesses of the defendant, that there had been a fracture of the neck of the femur, and that the head was now in the acetabulum.

The only remaining medical testimony called was Drs. Hopkins and Barton for plaintiff, and Dr. Miner, recalled for defendant.

Dr. Hopkins testified as follows: I reside in Buffalo, and am a practicing physician and surgeon. I know Bergman, the plaintiff. I first saw him about the twentieth of May, 1876, and at that time I made an examination of Bergman, as thoroughly as I could. I was not present at Bergman's house last Friday, but I saw him on the Sunday following. I made an examination, and in general terms determined his condition in presence of Drs. Gay, Barnes and Bartow. I would state that Dr. Gay asked me to see Bergman with him at that time. I examined Bergman to seek to account for the shortening of his limb. I was present at the operation of Dr. Gay in the attempt to reduce the dislocation. It would be improper for me to give an opinion on this case unless I am allowed to state what I observed at the time of the attempted reduction.

By the Court. Just tell the examination you made on Sunday last in the presence of these gentlemen.

We found Bergman with a shortening of the left leg of about two inches, and I was asked by Dr. Gay to account for this shortening. There were two theories which might account for it. The first was that the man was laboring under a dislocation, and the second was that he was suffering from a fracture. I first examined him to ascertain whether a fracture could account for his condition. We stripped the man, and laid him down upon the floor, upon just one comforter, and laid him flat upon his back for our examination. I found, upon measuring his left leg, that it was about two inches short, but to be as sure as possible, I called it one and a half inches, and I found the lower part of the back bent forward slightly. I found that as his left leg laid upon the floor you could pass you hand under it without touching it, from the knee to the hip. That it was raised up off the floor while the opposite leg lay upon the floor—from point to point it touched the floor. I then turned my attention to the manipulation of the limb. I

found that the head of the bone rotated very naturally—almost the same as the other side. I also found that by measuring from the anterior superior spinous process of the ilium to the trochanter, that it was a little shorter on the wounded side than on the other side. I turned him over on his belly. He objected at first and seemed not inclined to be troubled. I insisted, and he then allowed himself to be turned over. I then measured from fixed points on both sides, from the trochanters to the median line of the body. I made four measurements. I made a difference of three-quarters of an inch to one and one-eighth of and inch—the the wounded limb approached nearer to the median line of the body by about that much. I would also say that at a subsequent time I measured this man from the top of the trochanter to the tuberosity of the ischium, and I then found that on the injured side it was a little nearer, say three-quarters of an inch nearer than it was on the other side. From these facts I theorized in this way: In the shortening of the limb there are two ways to account for it, either the hip bone is out of place, or the neck has been broken and allowed the muscles to pull the shaft of the bone out of its place. These are the conditions which could account for that. I decided that the head of the bone was out of its place, for the following reasons: In dislocation into the sciatic notch, the head of the bone assumes nearly a natural position, as you will see it is nearly on the same line up and down (showing the bones). It makes a very fair socket. If the head of the bone is in the sciatic notch it makes a very good acetabulum, and the head will rotate. If the neck of the bone is fractured so as to allow the limb to shorten about two inches, this thing will result (showing the bones). We will draw from the centre of the neck a line two inches. In case of fracture of the neck of the femur, where the limb is shortened two, inches, and union takes place this thing must result. The neck of the bone comes down, and is attached to the shaft two inches below. From the shortening of the neck which always attends fracture of the neck, and its union in this new place free rotation is not possible. In case of union by ligament, with shortening of two inches, the head of the bone cannot be moved in its socket, and to take hold of the limb you can scarcely raise the limb from the floor but it will strike. You cannot rotate it or it will strike here (showing the bones that with two inches shortening the head would, if union occurred, be attached two inches lower than its natural position, and in turning the limb out the femur above would strike against the ilium). In such a case you might perhaps move it an inch this way or that way, but there is no possibility of anything like rotation. My point is, in a fracture, to account for two inches shortening, with union of the fractured neck, there would be no possibility of rotation, for the reasons I have given. It would be glued right on the bone, and you could not rotate it from its position. All the signs in the case point conclusively to a dislocation into the sciatic notch: the tipping up of the limb; the arch formed by the lower part of the spine; the mobility of the limb, showing it had a very good acetabulum, are the most positive and unequivocal signs of dislocation into the ischiatic notch. The approach of the tip of the trochanter towards the anterior spinous process about half an inch, towards the median line three-quarters of an inch, and towards the tuberosity of the ischium about say about three-quarters of an inch, are likewise all evidences. Professor Hamilton in his work on "Fractures and Dislocations" says: "Sir Astly Cooper remarks that this is the most difficult dislocation to detect and reduce, and Mr. Syme mentions a case in which the nature of the accident was overlooked by himself, and the thigh was not reduced until the thirteenth day, and subsequently Mr. Syme has called attention to what he considers as one of the most important diagnostic marks; indeed, he says it is never absent, nor is it ever met with in any other injury of the hip-joint, whether dislocation, fracture or bruise; this is an arched form of the lumbar part of the spine, which cannot be straightened so long as the thigh is straight, or on a line with the patient's trunk." (That is if you lay a man on his back and keep his thigh straight.) "When the limb is raised or bent upwards upon the pelvis, the back rests flat upon the bed; but so soon as the limb is allowed to descend, the back becomes arched as before." Dr. Hamilion says further: "That the trochanter major is approximated toward the anterior superior spinous process of the ilium." In Bergman's case I found the lumbar part of the spine arched, and I state that you diagnose between a fracture, and a dislocation into sciatic notch by taking the measurements which I have enumerated.

On cross-questioning, Dr. Hopkins said: That he had a notion since May 26th, the time of the operation, until last Sunday that the head might or might not be in place. Did not consider it necessary to back up Dr. Gay in his opinion of a successful reduction, on the contrary, Dr. Bartow and myself made up our minds that it was necessary to break Dr. Gay down in his opinion. We all changed opinion at the same time, the signs all indicate that he has sciatic dislocation. I might have said in conversation that the dislocation had been reduced, but do not remember having done so.

Dr. Bartow was called by the plaintiff. His testimony was chiefly coroborative of that of Dr. Hopkins with reference to there being sciatic dislocation. Said he had intimated in the interim between the twenty-sixth of May and Sunday last that the bone might not be in place, but Dr. Gay paid no attention to this suggestion.

Dr. Miner re-called. I have heard the testimony of Dr. Hopkins with reference to his opinion of the present lodgment of the head of the femur, and in which I understand him to say that he found the distance between the anterior superior spinous process of the ilium and the trochanter major to be one-half inch shorter on the injured side than the other. That agrees with my measurement. He also says that from the measurements which he made the trochanter is nearer the median line of the sacrum, and also the tuberosity of the ischium—so that he makes it nearer to three points at the same time which is mechanically impossible. There is but one possible condition in which it can be nearer the median line and also to the spinous process of the ilium, and that when the bone is broken and driven up—that is driven up or raised up nearer to both of these points This bone here is drawn up by the muscles whose force is very great. Something has been said in reference to it being on the sciatic notch, that is impossible. If it is out of the notch it would be drawn up upon the dorsum of the ilium. It is a mechanical impossiblity for it remain on the notch. It must be in the notch or drawn up on the dorsum of the ilium. With reference to rotation: If the head of the bone is in the notch the leg is turned in and the man walks with his foot turned in, and finds difficulty in getting the leg forward if he walks quickly. The toe is turned in on the dorsum of the other foot.

By Mr. Box. They speak about where there was a fracture of the femur, and where, if the bone should shove up, it would make some five inches shortening. Is there not such a thing as bony union ?

A. Where the neck was fractured, within the capsule, some surgeons formerly claimed that no bony union took place; but the ground is being abandoned. In numerous cases, I think, it is united by bone, and where united it is just as perfect, excepting short, and the ligaments are the same as in the normal limb. The leg is shortened, but the fracture does not prevent the movement upwards, outwards, inwards. The head is not carried down upon the shaft to the full amount of the shortening; the neck unites by bone or ligament in nearly its old position, the shortening is at the expense of the neck and not by the shaft being pushed up to any great extent—you have it all. We found it all perfect in this case. In no dislocation into the sciatic notch could he turn his leg out—it is entirely beyond the region of human possibilities.

Mr. L. L. Lewis summed up the case for the defence, Mr. Hubbell for the plaintiff, after which the charge was given to the jury by Judge Clinton.

The judge carefully charged the jury as to the responsibilities of a medical man, his duties to his patient in the case, etc., after which the court took a recess of fifteen minutes. At the conclusion of the recess the jury came in and rendered a verdict of "no cause of action." The clerk was directed to record a verdict for the defendant.

